# Social Jetlag on Obesity-Related Outcomes in Spanish Adolescents: Cross-Sectional Evidence from the EHDLA Study

**DOI:** 10.3390/nu16162574

**Published:** 2024-08-06

**Authors:** Mayra Fernanda Martínez-López, José Francisco López-Gil

**Affiliations:** 1 Cancer Research Group, Faculty of Medicine, Universidad de Las Américas, Quito 170124, Ecuador; 2 One Health Research Group, Universidad de Las Américas, Quito 170124, Ecuador; 3 Department of Communication and Education, Universidad Loyola Andalucía, 41704 Seville, Spain

**Keywords:** social jetlag, chronotype, youth, sleep duration, obesity, overweight, excess weight, abdominal obesity

## Abstract

Purpose: This study aimed to investigate the association between social jetlag (SJL) and obesity-related outcomes among adolescents from *Valle de Ricote* (Region of Murcia, Spain). We explored the relationship between SJL and body mass index (BMI) z-score, waist circumference, and body fat percentage, as well as the odds of having excess weight, obesity, and abdominal obesity in a sample of Spanish adolescents. Methods: A cross-sectional study was conducted using data from the Eating Healthy and Daily Life Activities (EHDLA) project, which included 847 Spanish adolescents aged 12–17 years. SJL was assessed based on the differences in sleep patterns between weekdays and weekends. Obesity-related indicators such as BMI z-score, waist circumference, body fat percentage, excess weight, obesity, and abdominal obesity were measured. Generalized linear models with a Gaussian or binomial distribution were used to analyze the associations between SJL and obesity-related outcomes, adjusting for potential confounders. Results: The analysis revealed significant associations between SJL and BMI z-score (unstandardized beta coefficient [*B*] = 0.15, 95% CI: 0.05 to 0.25, *p* = 0.003), waist circumference (*B* = 1.03, 95% CI: 0.39 to 1.67, *p* = 0.002), and body fat percentage (*B* = 0.83, 95% CI: 0.31 to 1.43, *p* = 0.008). Additionally, the odds ratios (ORs) for excess weight (OR = 1.35, 95% CI: 1.16 to 1.57; *p* < 0.001), obesity (OR = 1.59, 95% CI: 1.26 to 2.00; *p* < 0.001), and abdominal obesity (OR = 1.46, 95% CI: 1.23 to 1.72; *p* < 0.001) increased significantly with each 60 min increment in SJL. Conclusions: This study pointed out that the misalignment of sleeping times during weekdays and weekends (SJL) is significantly associated with higher BMI z-scores, waist circumference, body fat percentage, and higher odds of excess weight, obesity, and abdominal obesity among adolescents, being more significant in boys than in girls. These findings highlight the importance of addressing circadian misalignment in the prevention and management of obesity and its related metabolic disorders in this population.

## 1. Introduction

Adolescence is a transitional period marked by rapid physiological and psychosocial changes [1]. During this period, adolescents experience significant hormonal shifts that, along with their developing physiology and external factors such as social interactions, influence various behaviors [2]. One such behavior is the change in chronotype, which reflects an individual’s internal circadian rhythm, or “internal clock” [3]. During adolescence, the circadian rhythm shifts towards the later hours of the day, resulting in a preference for a later chronotype [4,5]. This shift means that adolescents’ natural sleep-wake timings often conflict with early morning obligations during the week, such as school schedules, that are typically designed to align with earlier chronotypes. This misalignment can lead to sleep deprivation and related issues, as adolescents struggle to reconcile their biological tendencies with societal demands [2,4]. To compensate for their altered circadian clock, adolescents often sleep past their usual waking hours on weekends, leading to a delay in their sleeping time, and generating a misalignment between work/weekdays and weekends [2,6]. This pattern, known as “social jetlag” (SJL), resembles the travel-induced jetlag caused by a three-day flight to the West [7]. Like travel-induced jetlag, SJL disrupts hormonal balance and physiology, leading to various detrimental effects on the body. It has been associated with increased levels of stress hormones, impaired cognitive function, and higher risks of chronic conditions such as obesity, diabetes, and cardiovascular diseases [8,9,10,11,12,13,14].

Childhood and adolescent obesity are becoming a rapidly escalating global issue, with over 160 million children and adolescents currently affected [15,16,17,18]. Initially classified as a first-world problem, the prevalence of adolescent obesity has reached alarming levels globally, affecting not just developed countries, but also low- and middle-income nations [18,19]. The prevalence of childhood and adolescent obesity has significantly risen in the past decades [18]. This rising epidemic poses significant health risks, including an increased likelihood of chronic diseases such as diabetes, cardiovascular issues, and mental health challenges [16,17,20]. It is therefore crucial to identify factors that influence the development of obesity in these susceptible populations to reduce the risk of developing comorbidities in adulthood.

The physiological and behavioral changes during adolescence, including shifts in circadian rhythm and the resulting SJL, contribute to the increasing prevalence of obesity in this age group. Sleep disturbances have been linked to weight gain and metabolic alterations [21]. Among these disturbances, SJL has shown a particularly significant impact on various adiposity-related variables beyond just BMI [21]. A study of 341 children aged 8–10 years old indicated that SJL is more strongly associated with increases in body fat percentage, fat mass, waist-to-hip ratio, and fat mass index compared to other sleep disturbances [22]. In adults, SJL is associated with a higher BMI, an increased waist circumference, elevated cholesterol levels, and a greater risk of developing morbid obesity in individuals who have already obesity [13,23]. In addition, high levels of SJL have a significant effect on increasing systolic blood pressure and glycated hemoglobin levels, which are key indicators of metabolic and cardiovascular health [9,13]. To support these findings, a meta-analysis that evaluated 43 studies with a sample size of 231,648 participants showed that SJL was positively and consistently associated with multiple obesity-related anthropometric measures, including BMI, fat mass, fat mass index, body fat percentage, waist circumference, and the risk of having overweight/obesity [24].

On the other hand, chronotype influences food choices, as shown in a previous study among 820 Spanish adolescents, where those with later chronotypes had a lower probability of adhering to the Mediterranean diet, which is known to be highly beneficial for metabolic and cardiovascular health [25], compared to earlier chronotypes [26]. Due to the strong relationship between later chronotypes and SJL in adolescents, it is expected that SJL may affect dietary choices. For instance, a cross-sectional study involving 710 participants aged 19–24 years found that individuals with pronounced SJL had a higher total energy intake, including an increased consumption of saturated fats and overall unhealthy foods within a short time window compared to those without SJL [27]. Similarly, in a study of 3567 Chinese adolescents, it was shown that SJL correlated with the increased daily consumption of unhealthy foods [28]. Additionally, a study analyzing a sample of 2985 children pointed out that greater SJL resulted in a higher consumption of fast food and sweetened drinks and a lower consumption of fruits and vegetables [29]. This effect is also observed in adults, where high SJL was associated with poorer diet choices [30], unhealthy eating behaviors, higher alcohol consumption, and lower amounts of physical activity [31,32]. Furthermore, these studies indicated that higher SJL was correlated with greater obesity measures such as BMI, highlighting the broad impact of SJL on dietary habits and body weight [27,29,30,31]. The effects of SJL on diet and weight are more pronounced in individuals with obesity-related chronic conditions such as type 2 diabetes (DM2). For instance, in a study of 792 Brazilian adults, patients with SJL and a pre-existing diagnosis of DM2 reported a higher energy intake past 9 pm and made fewer healthy food choices [32]. These observations were supported in preclinical settings by animal studies. Specifically, when rats were exposed to unhealthy diets, those with SJL were more likely to overeat and develop metabolic syndrome [33]. Importantly, while SJL might not directly influence weight gain in individuals who follow a healthy diet, it has a high likelihood of altering their metabolic parameters [10,13,32]. This suggests that although a healthy diet may mitigate the direct impact of SJL on weight gain, SJL can still predispose individuals to metabolic disturbances, especially when combined with poor dietary habits.

Given the alarming global rise in childhood and adolescent obesity and its associated health risks, it is crucial to identify factors contributing to this epidemic, particularly in vulnerable populations. Most existing studies focus on diverse, more global demographics (i.e., China and the USA, among others), potentially overlooking how SJL impacts specific, smaller communities that may have distinct cultural, dietary, and lifestyle patterns. Therefore, this study assessed the association of SJL with obesity-related outcomes in adolescents from *Valle de Ricote* (Murcia, Spain), a region with unique cultural practices that influence daily diet and lifestyle [34], as well as with a high prevalence of excess weight among children and adolescents [35,36,37,38]. Additionally, this study examines various obesity-related outcomes such as BMI z-score, waist circumference, body fat percentage, and the odds of excess weight, obesity, and abdominal obesity, which are often not comprehensively covered in other studies that tend to focus on a narrower set of obesity indicators. Furthermore, a significant gap in the existing research is the insufficient control for potential confounders (i.e., sex, age, socioeconomic status, physical activity, sedentary behavior, adherence to the Mediterranean diet, and energy intake) that could influence the relationship between SJL and obesity. This approach strengthens the validity and applicability of our findings, providing a clearer picture of how SJL directly impacts obesity measures.

## 2. Materials and Methods

### 2.1. Study Design and Population

The cross-sectional study conducted for this research utilized data from the Eating Healthy and Daily Life Activities (EHDLA) project, whose protocol is already published [39]. The study comprised Spanish adolescents aged 12–17 years from three secondary schools in *Valle de Ricote*, Region of Murcia, Spain, and the data were collected during the 2021–2022 academic year. Initially, there were 1378 adolescents (100%) in the EHDLA study, but 119 (8.7%) were excluded due to missing anthropometric data. Further exclusions were made for participants lacking information on overall meal duration (*n* = 464; 33.7%), overall sleep duration (*n* = 33; 2.4%), and physical activity (*n* = 7; 0.5%). Ultimately, 847 adolescents (45.2% boys) were included in the study. Obtaining written consent from parents or guardians was required for all participants, and adolescents were informed about the study’s objectives and provided their consent. This research was approved by the Bioethics Committee of the University of Murcia and the Ethics Committee of the Albacete University Hospital Complex, as well as the Albacete Integrated Care Management (approval IDs: 2218/2018 and 2021-85, respectively). This study adhered to the ethical principles outlined in the Helsinki Declaration.

### 2.2. Procedures

#### 2.2.1. Social Jetlag (Independent Variable)

SJL was assessed by collecting self-reported bedtimes and wake times for both weekdays and weekends. The midpoint of sleep was calculated, and SJL was defined as the absolute difference between sleep onset times on weekdays and weekends [7,40].

#### 2.2.2. Obesity-Related Indicators (Dependent Variables)

We utilized electronic scales (Tanita BC-545, Tokyo, Japan) and portable height rods (Leicester Tanita HR 001, Tokyo, Japan) to measure body weight and height, respectively. BMI was calculated by dividing weight in kilograms by the square of height in meters. We determined the BMI z-score using World Health Organization (WHO) age-specific and sex-specific thresholds [41]. To assess excess weight (including overweight and obesity), we used these BMI z-scores. Waist circumference was measured at the level of the umbilicus using a constant tension tape, and the waist-to-height ratio (WHtR) was calculated, with a value of ≥0.5 indicating abdominal obesity [42]. Skinfold measurements at the triceps and medial calf were taken using calibrated steel calipers (Holtain, Crosswell, Crymych, United Kingdom) [43]. We estimated body fat percentage using the Slaughter et al. equations for children and adolescents [44].

#### 2.2.3. Covariates

Participants provided their sex and age. Socioeconomic status was evaluated using the Family Affluence Scale (FAS-III), which involves summing responses to questions about family possessions and amenities [45]. Physical activity and sedentary behavior were assessed using the Youth Activity Profile (YAP) questionnaire [46]. Adherence to the Mediterranean Diet was measured using the Mediterranean Diet Quality Index for Children and Adolescents (KIDMED) [47]. Energy intake was calculated using a self-administered food frequency questionnaire validated for the Spanish population [48].

### 2.3. Statistical Analysis

Data were presented as absolute and relative numbers (percentages) for categorical variables and as medians and interquartile ranges (IQRs) for continuous variables. Generalized linear models (GLMs) with Gaussian distribution were used for continuous outcomes and GLMs with binomial distribution were used for dichotomous outcomes. These analyses were conducted including both boys and girls and were stratified by sex. SJL was the primary independent variable, measured in 60 min increments. Models were adjusted for potential confounders, including age, sex, socioeconomic status, physical activity, sedentary behavior, adherence to the Mediterranean diet, and energy intake. The results were presented as unstandardized beta coefficients (*B*) for continuous outcomes and odds ratios (ORs) for dichotomous outcomes, with standard errors (SEs) and 95% confidence intervals (CIs). Estimated marginal means for BMI z-score, waist circumference, and body fat percentage, and predictive probabilities for excess weight, obesity, and abdominal obesity, were computed in relation to SJL. A post-hoc power analysis was conducted using a small effect size (Cohen’s *d* = 0.20), a sample size of 847, and a significance level of 0.05. The analysis indicated a power of approximately 98.4%, suggesting that the study had sufficient power to detect small effect sizes, thereby supporting the reliability of the study’s findings. Statistical analyses were conducted using R software (version 4.4.1) and RStudio (version 2024.04.2+764). A *p*-value of less than 0.050 was considered statistically significant.

## 3. Results

Table 1 provides a comprehensive overview of the descriptive data of study participants. The median SJL was 90 min (IQR = 90). Furthermore, the median BMI z-score was 0.0 (IQR = 2.0). The median waist was 70.9 cm (IQR = 13.3), and the median body fat percentage was 23.7% (IQR = 12.4). Of the study participants, 25.6% had excess weight and 8.9% had obesity. Abdominal obesity was present in 20.3% of the participants.

Table 2 presents the GLM assessing the association between SJL (per 10 min) and various obesity-related outcomes. The analysis revealed significant associations between SJL and BMI z-score (*B* = 0.15, 95% CI: 0.05 to 0.25, *p* = 0.003), waist circumference (*B* = 1.03, 95% CI: 0.39 to 1.67, *p* = 0.002), and body fat percentage (*B* = 0.83, 95% CI: 0.31 to 1.43, *p* = 0.008). Additionally, the ORs for excess weight (OR = 1.35, 95% CI: 1.16 to 1.57; *p* < 0.001), obesity (OR = 1.59, 95% CI: 1.26 to 2.00; *p* < 0.001), and abdominal obesity (OR = 1.46, 95% CI: 1.23 to 1.72; *p* < 0.001) increased significantly with each 60 min increment in SJL. Additional analyses, detailed in Tables S1–S6, showed the complete results of the associations of SJL and covariates with obesity-related outcomes. Furthermore, to explore potential differences in the association between SJL and obesity-related outcomes by sex, stratified analyses are found in Tables S7 and S8.

**Figure 1:**
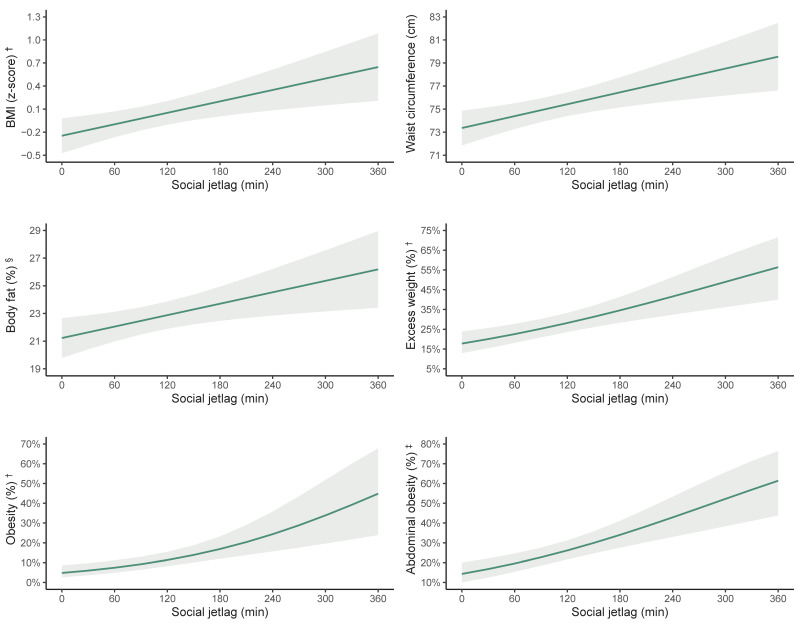
Estimated marginal means and predictive probabilities for the different obesity-related outcomes based on social jetlag among Spanish adolescents. BMI, body mass index. ^†^ According to the World Health Organization criteria [41]. ^§^ According to the Slaughter et al. equations [44]. ^‡^ Using a cutoff point of waist-to-height ratio ≥ 0.5 [42].

Figure 1 provides a graphical representation of the relationship between SJL and various obesity-related outcomes. The graphs show the positive association between social jetlag (in minutes) and BMI z-score, waist circumference, body fat percentage, excess weight percentage, obesity percentage, and abdominal obesity percentage. These visual representations further highlight the trend in increasing obesity-related outcomes with greater SJL.

## 4. Discussion

The present study investigated the association between SJL and obesity-related outcomes among Spanish adolescents from *Valle de Ricote* (Region of Murcia, Spain). Here, we found a significant association between higher SJL and higher BMI z-scores, waist circumference, and body fat percentage. Additionally, adolescents with a greater SJL had higher odds of excess weight, obesity, and abdominal obesity. These results align with previous research suggesting that circadian misalignment, particularly SJL, could contribute to adverse metabolic outcomes (e.g., higher BMI, adiposity, waist circumference, odds of having excess weight) [24]. For instance, recent studies found that adolescents and young adults with higher SJL levels had a higher BMI, fat mass, and waist circumference [49,50], reinforcing that the disruption of biological and social time can lead to obesity and metabolic syndrome. Similarly, other studies have shown that children and adolescents with greater SJL levels are more likely to exhibit higher body fat percentages and waist-to-hip ratios [11,22,28,29,31,51,52,53,54,55], supporting the notion that SJL negatively impacts adiposity and overall health.

Adolescents experiencing SJL often have irregular sleep patterns, leading to alterations in their circadian rhythms [56]. This can significantly impact the regulation of critical appetite-related hormones, including ghrelin and leptin, which play vital roles in hunger and satiety [49,57]. Ghrelin, which promotes hunger, is primarily secreted by the stomach and signals hunger to the brain, thereby increasing appetite [58]. Opposite to ghrelin, the adipose tissue secretes leptin, which helps to suppress appetite by signaling to the brain that the body has enough energy stores [59,60]. Under normal conditions, these hormones work in a balanced manner to regulate food consumption and energy expenditure [61,62]. However, when circadian rhythms are disrupted due to irregular sleep patterns, as seen in SJL, the balance of these hormones is adversely affected [63,64,65,66,67]. More specifically, it was pointed out that partial sleep deprivation led to a significant decrease in leptin levels and an increase in ghrelin levels, which together promote hunger and increased energy intake [68,69,70].

In addition to leptin and ghrelin, other physiological factors may influence the impact of SJL in obesity, such as insulin sensitivity, cortisol secretion, intestinal peptides, and alterations in the autonomous nervous system [71,72]. Research has shown that individuals with greater SJL often exhibit signs of metabolic disturbances, including insulin resistance and elevated cortisol levels, which can promote fat accumulation, particularly in the abdominal region [11,73]. Abdominal obesity is a critical indicator of metabolic syndrome and is associated with increased risks of DM2, cardiovascular diseases, and other metabolic disorders [74]. Specifically, one study by Mota et al. [75] identified that adults with greater SJL had higher insulin resistance and cortisol levels, which are key indicators of metabolic syndrome. Furthermore, disruptions in melatonin secretion, a hormone that regulates sleep–wake cycles, have been linked to an increased risk of obesity and metabolic disorders [76,77]. Adolescents with SJL may experience lower nocturnal melatonin levels, which can exacerbate metabolic dysregulation [55].

Additionally, adolescents with high SJL levels often engage in unhealthy behaviors, such as irregular meal patterns, late-night eating, and reduced physical activity [26,51,78,79]. These irregularities can disrupt metabolic processes and lead to excessive energy intake. Staying up late and waking up late also limits opportunities for physical exercise, contributing to a sedentary lifestyle, a well-known risk factor for obesity [80]. Sleep deprivation can also lead to the increased consumption of high-calorie, sugary, and fatty foods as a compensatory mechanism to combat fatigue and enhance mood [69,81,82]. This dietary behavior, coupled with reduced physical activity due to tiredness, can contribute to obesity [83,84]. In this regard, previous research has shown that SJL is associated with a poorer diet quality [26,27,28,29,30,32,85]. Adolescents with higher SJL had lower KIDMED scores, indicating poorer adherence to the Mediterranean diet. This dietary pattern, known for its benefits in metabolic health and weight management, includes a high consumption of fruits, vegetables, whole grains, and healthy fats, and a low consumption of ultra-processed foods and sugars [25]. Adolescents with higher SJL levels tend to consume more high-calorie, low-nutrient foods, contributing to weight gain and metabolic disturbances [26,28,29,51,53]. The disruption in meal timing and composition can exacerbate insulin resistance and increase cortisol levels, further promoting central adiposity and metabolic syndrome [3,10,23].

On the other hand, although our results showed similar patterns in both boys and girls, the association between SJL and the presence of cardiometabolic comorbidities in adolescents could be different in relation to sex [86]. In fact, in the stratified analyses, the associations seem to be stronger for boys than girls. Conversely, in a study of 278 healthy adolescents aged 9–15 years, only girls with high SJL showed indicators of adverse cardiometabolic profiles, such as higher total cholesterol, high-density lipoproteins cholesterol (HDL-c) ratio, and systolic blood pressure [11]. These associations were not observed in boys with high SJL [11]. However, no significant associations to obesity-related outcomes were identified for either boys or girls. Along the same line, high SJL levels showed a positive association with higher BMI (z-score) in girls but not in boys within a study including 381 children aged 9–11 years [53]. These discrepancies could be explained by the inclusion of different covariates in our analyses that were absent in the other two studies, including lifestyle factors (e.g., physical activity [11], sedentary behavior [11,53], and energy intake [11,53]) and sociodemographic variables (e.g., socioeconomic status [11,53]). Furthermore, adolescent boys may be more affected by social jet lag than girls due to several factors, including biological tendencies toward evening chronotypes, higher testosterone levels, and greater participation in evening activities [50,87,88]. These factors can contribute to a more significant desynchronization between their internal clocks and social schedules [50,88]. Additionally, social and cultural pressures may further exacerbate these differences [89,90]. Thus, more studies are needed to fully elucidate the relationship between sex and SJL in relation to obesity-related indicators.

This study has several limitations that should be considered. Firstly, the cross-sectional design precludes establishing causal relationships between SJL and obesity-related outcomes, underscoring the need for longitudinal studies to clarify the temporal relationship between SJL and metabolic health. Secondly, the reliance on self-reported data for sleep patterns, physical activity, and energy intake may introduce reporting biases. Objective measures, such as actigraphy for sleep assessment and detailed dietary recalls, would provide more accurate evaluations. Thirdly, the study population was limited to adolescents from the *Valle de Ricote* (Region of Murcia, Spain), potentially affecting the generalizability of the findings to other populations. Despite these limitations, this study also has notable strengths. It includes a large sample size and uses objective measures for anthropometric assessments, enhancing the reliability of the data. The comprehensive analysis of various obesity-related outcomes, including BMI z-scores, waist circumference, and body fat percentage, provides robust evidence of the impact of SJL on metabolic health in adolescents. Additionally, we controlled for potential confounders such as energy intake, which allowed for a more objective assessment of the effects of SJL on food intake and consequent weight gain and obesity. These strengths contribute valuable insights into the relationship between SJL and obesity-related outcomes among adolescents.

## 5. Conclusions

This study pointed out that SJL is significantly associated with higher BMI z-scores, waist circumference, body fat percentage, and higher odds of excess weight, obesity, and abdominal obesity among adolescents, being more significant in boys than in girls. These findings highlight the importance of addressing circadian misalignment in the prevention and management of obesity and its related metabolic disorders in this population. Future research should explore the underlying mechanisms linking SJL to metabolic health and evaluate the effectiveness of interventions aimed at reducing SJL to improve metabolic outcomes. More longitudinal studies are needed to establish causal relationships between SJL and metabolic health outcomes. Additionally, investigating the relationship between SJL, dietary habits, physical activity, and other lifestyle factors will provide a more comprehensive understanding of the pathways through which SJL influences obesity and metabolic health. Interventions targeting SJL should be developed and tested to determine their effectiveness in improving metabolic health and preventing obesity in adolescents.

## Figures and Tables

**Table 1:** Descriptive data of the study participants (*N* = 847).

Variable		Total
Age (years)	Median (IQR)	14.0 (2.0)
Sex	Boys (%)	379 (44.7)
	Girls (%)	468 (55.3)
FAS-III (score)	Median (IQR)	8.0 (3.0)
YAP-S physical activity global (score)	Median (IQR)	2.6 (0.8)
YAP-S sedentary behaviors (score)	Median (IQR)	2.6 (0.8)
KIDMED (score)	Median (IQR)	7.0 (3.0)
Energy intake (kcal)	Median (IQR)	2589.3 (1490.3)
Social jetlag (min)	Median (IQR)	90.0 (90.0)
BMI (z-score) ^†^	Median (IQR)	0.0 (2.0)
Waist circumference (cm)	Median (IQR)	70.9 (13.3)
Body fat (%) ^§^	Median (IQR)	23.7 (12.4)
Excess weight ^†^	No (%)	630 (74.4)
	Yes (%)	217 (25.6)
Obesity ^†^	No (%)	772 (91.1)
	Yes (%)	75 (8.9)
Abdominal obesity ^‡^	No (%)	675 (79.7)
	Yes (%)	172 (20.3)

BMI, body mass index; FAS-III, Family Affluence Scale-III; IQR, interquartile range; KIDMED, Mediterranean Diet Quality Index for Children and Adolescents; YAP-S, Spanish Youth Active Profile. ^†^ According to the World Health Organization criteria [41]. ^§^ According to the Slaughter et al. equations [44]. ^‡^ Using a cutoff point of waist-to-height ratio ≥ 0.5 [42].

**Table 2:** Generalized linear model assessing the association between social jetlag and different obesity-related outcomes among adolescents.

	**Independent variable: social jetlag (per 60 min)**				
Dependent variables (continuous)	*B*	SE	LLCI	ULCI	*p*-value
BMI (z-score) ^†^	0.15	0.05	0.05	0.25	0.003
Waist circumference (cm)	1.03	0.33	0.39	1.67	0.002
Body fat (%) ^§^	0.83	0.31	0.22	1.43	0.008
Dependent variables (dichotomic)	OR	SE	LLCI	ULCI	*p*-value
Excess weight (yes) ^†^	1.35	0.08	1.16	1.57	<0.001
Obesity (yes) ^†^	1.59	0.12	1.26	2.00	<0.001
Abdominal obesity (yes) ^‡^	1.46	0.08	1.23	1.72	<0.001

Adjusted for age, sex, socioeconomic status, physical activity, sedentary behavior, adherence to the Mediterranean diet, and energy intake. *B*, unstandardized beta coefficient; BMI, body mass index; LLCI, lower limit confidence interval; OR, odds ratio; SE, standard error; ULCI, upper limit confidence interval. ^†^ According to the World Health Organization criteria [41]. ^§^ According to the Slaughter et al. equations [44]. ^‡^ Using a cutoff point of waist-to-height ratio ≥ 0.5 [42].

## Data Availability

The data used in this study are available upon request from the corresponding authors. However, given that the participants are minors, privacy and confidentiality must be respected.

## Supplementary Materials

The following supporting information can be downloaded at: https://www.mdpi.com/article/10.3390/nu16162574/s1, Table S1: Generalized linear model assessing the association between social jetlag and body mass index z-score ^†^ among adolescents; Table S2: Generalized linear model assessing the association between social jetlag and waist circumference among adolescents; Table S3: Generalized linear model assessing the association between social jetlag and body fat percentage ^§^ among adolescents; Table S4: Generalized linear model assessing the association between social jetlag and excess weight ^†^ among adolescents; Table S5: Generalized linear model assessing the association between social jetlag and obesity ^†^ among adolescents; Table S6: Generalized linear model assessing the association between social jetlag and abdominal obesity ^‡^ among adolescents; Table S7: Generalized linear model assessing the association between social jetlag and different obesity-related outcomes among male adolescents; Table S8: Generalized linear model assessing the association between social jetlag and different obesity-related outcomes among female adolescents.
