# Supplementary material for: Social Jetlag on Obesity-Related Outcomes in Spanish Adolescents: Cross-Sectional Evidence from the EHDLA Study
Source: Nutrients. 2024 Aug 6;16(16):2574. doi: 10.3390/nu16162574 (PMC11357624; doi:10.3390/nu16162574)
Supplement: Supplementary file 1 [file nutrients-16-02574-s001.zip › nutrients-3135195-supplementary.pdf]

Supplementary Material

Table S1. Generalized linear model assessing the association between social jetlag and body mass index z-score † among adolescents.

Predictor	*B*	SE	LLCI	ULCI	*p*-value
Social jetlag (per 60 min)	0.15	0.05	0.05	0.25	0.003
Age (per one year)	0.10	0.03	0.03	0.17	0.004
Sex					
Boys	Reference				
Girls	0.08	0.10	-0.13	0.28	0.457
FAS-III (per one point)	-0.04	0.02	-0.09	0.01	0.099
YAP-S physical activity (per one point)	-0.01	0.08	-0.16	0.15	0.910
Overall sleep duration (per one hour)	0.01	0.06	-0.11	0.13	0.917
YAP-S sedentary behaviors (per one point)	0.19	0.09	0.01	0.36	0.035
KIDMED (per one point)	0.02	0.02	-0.02	0.06	0.424
Energy intake (per 1000 kcal)	-0.04	0.03	-0.10	0.01	0.090

B, unstandardized beta coefficient; FAS-III; Family Affluence Scale-III; KIDMED, Mediterranean Diet Quality Index for Children and Adolescents; LLCI, lower limit confidence interval; SE, standard error; ULCI, upper limit confidence interval; YAP-S, Spanish Youth Active Profile. † According to the World Health Organization criteria [35].

Table S2. Generalized linear model assessing the association between social jetlag and waist circumference among adolescents.

Predictor	*B*	SE	LLCI	ULCI	*p*-value
Social jetlag (per 60 min)	1.03	0.33	0.39	1.67	0.002
Age (per one year)	0.78	0.23	0.32	1.23	0.001
Sex					
Boys	Reference				
Girls	-4.90	0.70	-6.28	-3.53	<0.001
FAS-III (per one point)	-0.16	0.16	-0.47	0.15	0.304
YAP-S physical activity (per one point)	0.27	0.53	-0.76	1.30	0.609
Overall sleep duration (per one hour)	-0.20	0.41	-1.01	0.60	0.620
YAP-S sedentary behaviors (per one point)	0.94	0.59	-0.22	2.10	0.112
KIDMED (per one point)	0.15	0.14	-0.13	0.43	0.301
Energy intake (per 1000 kcal)	-0.16	0.17	-0.50	0.17	0.342

B, unstandardized beta coefficient; FAS-III; Family Affluence Scale-III; KIDMED, Mediterranean Diet Quality Index for Children and Adolescents; LLCI, lower limit confidence interval; SE, standard error; ULCI, upper limit confidence interval; YAP-S, Spanish Youth Active Profile.

Table S3. Generalized linear model assessing the association between social jetlag and body fat percentage § among adolescents.

Predictor	*B*	SE	LLCI	ULCI	*p*-value
Social jetlag (per 60 min)	0.83	0.31	0.22	1.43	0.008
Age (per one year)	-0.59	0.22	-1.02	-0.16	0.007
Sex					
Boys	Reference				
Girls	2.90	0.67	1.59	4.22	<0.001
FAS-III (per one point)	-0.10	0.15	-0.39	0.20	0.526
YAP-S physical activity (per one point)	-0.35	0.50	-1.34	0.63	0.482
Overall sleep duration (per one hour)	0.56	0.39	-0.21	1.33	0.155
YAP-S sedentary behaviors (per one point)	1.01	0.56	-0.10	2.12	0.074
KIDMED (per one point)	0.04	0.14	-0.23	0.31	0.764
Energy intake (per 1000 kcal)	-0.29	0.17	-0.62	0.04	0.083

B, unstandardized beta coefficient; FAS-III; Family Affluence Scale-III; KIDMED, Mediterranean Diet Quality Index for Children and Adolescents; LLCI, lower limit confidence interval; SE, standard error; ULCI, upper limit confidence interval; YAP-S, Spanish Youth Active Profile. § According to the Slaughter et al. equations [38].

Table S4. Generalized linear model assessing the association between social jetlag and excess weight † among adolescents.

Predictor	*B*	SE	LLCI	ULCI	*p*-value
Social jetlag (per 60 min)	1.35	0.08	1.16	1.57	<0.001
Age (per one year)	1.05	0.06	0.94	1.17	0.426
Sex					
Boys	Reference				
Girls	0.82	0.17	0.59	1.15	0.257
FAS-III (per one point)	0.96	0.04	0.89	1.04	0.298
YAP-S physical activity (per one point)	1.11	0.13	0.87	1.43	0.400
Overall sleep duration (per one hour)	1.16	0.14	0.88	1.53	0.289
YAP-S sedentary behaviors (per one point)	0.95	0.10	0.78	1.16	0.615
KIDMED (per one point)	1.05	0.04	0.98	1.13	0.162
Energy intake (per 1000 kcal)	0.88	0.06	0.79	0.99	0.027

B, unstandardized beta coefficient; FAS-III; Family Affluence Scale-III; KIDMED, Mediterranean Diet Quality Index for Children and Adolescents; LLCI, lower limit confidence interval; SE, standard error; ULCI, upper limit confidence interval; YAP-S, Spanish Youth Active Profile. † According to the World Health Organization criteria [35].

Table S5. Generalized linear model assessing the association between social jetlag and obesity † among adolescents.

Predictor	*B*	SE	LLCI	ULCI	*p*-value
Social jetlag (per 60 min)	1.59	0.12	1.26	2.00	<0.001
Age (per one year)	1.10	0.09	0.92	1.33	0.305
Sex					
Boys	Reference				
Girls	0.46	0.29	0.26	0.83	0.009
FAS-III (per one point)	0.96	0.06	0.85	1.09	0.573
YAP-S physical activity (per one point)	0.89	0.22	0.58	1.37	0.593
Overall sleep duration (per one hour)	1.18	0.23	0.76	1.83	0.467
YAP-S sedentary behaviors (per one point)	0.75	0.16	0.55	1.03	0.073
KIDMED (per one point)	1.02	0.06	0.90	1.14	0.793
Energy intake (per 1000 kcal)	1.00	0.07	0.87	1.14	0.971

B, unstandardized beta coefficient; FAS-III; Family Affluence Scale-III; KIDMED, Mediterranean Diet Quality Index for Children and Adolescents; LLCI, lower limit confidence interval; SE, standard error; ULCI, upper limit confidence interval; YAP-S, Spanish Youth Active Profile. † According to the World Health Organization criteria [35].

Table S6. Generalized linear model assessing the association between social jetlag and abdominal obesity ‡ among adolescents.

Predictor	*B*	SE	LLCI	ULCI	*p*-value
Social jetlag (per 60 min)	1.46	0.08	1.23	1.72	<0.001
Age (per one year)	0.94	0.06	0.83	1.07	0.338
Sex					
Boys	Reference				
Girls	0.53	0.19	0.37	0.77	0.001
FAS-III (per one point)	0.93	0.04	0.85	1.01	0.068
YAP-S physical activity (per one point)	1.05	0.14	0.80	1.39	0.719
Overall sleep duration (per one hour)	1.04	0.16	0.76	1.41	0.813
YAP-S sedentary behaviors (per one point)	0.92	0.11	0.74	1.15	0.476
KIDMED (per one point)	1.01	0.04	0.93	1.09	0.881
Energy intake (per 1000 kcal)	0.88	0.07	0.77	1.00	0.042

B, unstandardized beta coefficient; FAS-III; Family Affluence Scale-III; KIDMED, Mediterranean Diet Quality Index for Children and Adolescents; LLCI, lower limit confidence interval; SE, standard error; ULCI, upper limit confidence interval; YAP-S, Spanish Youth Active Profile. ‡ Using a cut-off point of waist-to-height ratio ≥ 0.5 [36].

Table S7. Generalized linear model assessing the association between social jetlag and different obesity-related outcomes among male adolescents.

	Independent variable: Social jetlag (per 60 min)				
Dependent variables (continuous)	*B*	SE	LLCI	ULCI	*p*-value
Body mass index (z-score) †	0.23	0.08	0.07	0.39	0.006
Waist circumference (cm)	1.88	0.55	0.80	2.96	0.001
Body fat (%) §	1.42	0.55	0.35	2.50	0.010
Dependent variables (dichotomic)	OR	SE	LLCI	ULCI	*p*-value
Excess weight (yes) †	1.49	0.11	1.19	1.85	<0.001
Obesity (yes) †	1.83	0.16	1.34	2.51	<0.001
Abdominal obesity (yes) ‡	1.57	0.12	1.25	1.97	<0.001

Adjusted for age, socioeconomic status, physical activity, sedentary behavior, adherence to the Mediterranean diet, and energy intake. *B*, unstandardized beta coefficient; LLCI, lower limit confidence interval; OR, odds ratio; SE, standard error; ULCI, upper limit confidence interval. † According to the World Health Organization criteria [35]. § According to the Slaughter et al. equations [38]. ‡ Using a cut-off point of waist-to-height ratio ≥ 0.5 [36].

Table S8. Generalized linear model assessing the association between social jetlag and different obesity-related outcomes among female adolescents.

	Independent variable: Social jetlag (per 60 min)				
Dependent variables (continuous)	*B*	SE	LLCI	ULCI	*p*-value
Body mass index (z-score) †	0.08	0.06	-0.03	0.20	0.157
Waist circumference (cm)	0.53	0.40	-0.24	1.31	0.176
Body fat (%) §	0.36	0.33	-0.30	1.01	0.287
Dependent variables (dichotomic)	OR	SE	LLCI	ULCI	*p*-value
Excess weight (yes) †	1.29	0.11	1.03	1.61	0.024
Obesity (yes) †	1.16	0.20	0.78	1.71	0.457
Abdominal obesity (yes) ‡	1.27	0.13	0.98	1.64	0.065

Adjusted for age, socioeconomic status, physical activity, sedentary behavior, adherence to the Mediterranean diet, and energy intake. *B*, unstandardized beta coefficient; LLCI, lower limit confidence interval; OR, odds ratio; SE, standard error; ULCI, upper limit confidence interval. † According to the World Health Organization criteria [35]. § According to the Slaughter et al. equations [38]. ‡ Using a cut-off point of waist-to-height ratio ≥ 0.5 [36].
